# Dual soluble epoxide hydrolase inhibitor – farnesoid X receptor agonist interventional treatment attenuates renal inflammation and fibrosis

**DOI:** 10.3389/fimmu.2023.1269261

**Published:** 2024-01-03

**Authors:** Md. Abdul Hye Khan, Benjamin Nolan, Anna Stavniichuk, Daniel Merk, John D. Imig

**Affiliations:** ^1^ Drug Discovery Center, Medical College of Wisconsin, Milwaukee, WI, United States; ^2^ Department of Integrative Biology and Pharmacology, The University of Texas Health Science Center at Houston, Houston, TX, United States; ^3^ Department of Pharmacy, Ludwig-Maximilians Universität München, Munich, Germany; ^4^ Department of Pharmaceutical Sciences, University of Arkansas for Medical Sciences, Little Rock, AR, United States

**Keywords:** dual acting molecule, farnesoid X receptor agonist, soluble epoxide hydrolase, inflammation kidney, fibrosis, chronic kidney disease

## Abstract

**Introduction:**

Renal fibrosis associated with inflammation is a critical pathophysiological event in chronic kidney disease (CKD). We have developed DM509 which acts concurrently as a farnesoid X receptor agonist and a soluble epoxide hydrolase inhibitor and investigated DM509 efficacy as an interventional treatment using the unilateral ureteral obstruction (UUO) mouse model.

**Methods:**

Male mice went through either UUO or sham surgery. Interventional DM509 treatment (10mg/kg/d) was started three days after UUO induction and continued for 7 days. Plasma and kidney tissue were collected at the end of the experimental protocol.

**Results:**

UUO mice demonstrated marked renal fibrosis with higher kidney hydroxyproline content and collagen positive area. Interventional DM509 treatment reduced hydroxyproline content by 41% and collagen positive area by 65%. Renal inflammation was evident in UUO mice with elevated MCP-1, CD45-positive immune cell positive infiltration, and profibrotic inflammatory gene expression. DM509 treatment reduced renal inflammation in UUO mice. Renal fibrosis in UUO was associated with epithelial-to-mesenchymal transition (EMT) and DM509 treatment reduced EMT. UUO mice also had tubular epithelial barrier injury with increased renal KIM-1, NGAL expression. DM509 reduced tubular injury markers by 25-50% and maintained tubular epithelial integrity in UUO mice. Vascular inflammation was evident in UUO mice with 9 to 20-fold higher ICAM and VCAM gene expression which was reduced by 40-50% with DM509 treatment. Peritubular vascular density was reduced by 35% in UUO mice and DM509 prevented vascular loss.

**Discussion:**

Interventional treatment with DM509 reduced renal fibrosis and inflammation in UUO mice demonstrating that DM509 is a promising drug that combats renal epithelial and vascular pathological events associated with progression of CKD.

## Introduction

Renal fibrosis associated with inflammation is considered as the final common pathway by which chronic kidney disease (CKD) leads to end stage renal disease (ESRD) (1, 2). Occurrence of CKD and ESRD are very common in patients with inflammatory diseases such as cardiometabolic syndrome, diabetes, and hypertension (2–4). These are the most common chronic diseases of the modern world that cause CKD and high mortality (3, 4). The high mortality and morbidity associated with CKD often linked to the lack of an effective anti-fibrotic and anti-inflammatory agents that could target renal fibrosis (4).

There is an unmet need of novel combined anti-inflammatory and anti-fibrotic agents, particularly as an effective therapeutic approach for CKD and its progression to ESRD (5–7). Angiotensin-converting enzyme inhibitors and angiotensin II receptor blockers are the current therapeutic choices for the clinical management and treatment of CKD (8). However, these mainstay treatment options have limited efficacy to prevent or treat renal fibrosis, the major pathological event that leads to the progression of CKD to ESRD (5, 6). More recently, finerenone, a nonsteroidal selective mineralocorticoid receptor antagonist, has been demonstrated to decrease albuminuria and risk of CKD progression in chronic heart failure and diabetes (9, 10). Other novel approaches that have been attempted include the nuclear factor E2-related factor 2 inducer bardoxolone and the endothelin receptor blocker avosentan which proved largely ineffective in treating inflammation and associated renal fibrosis (11, 12). Findings from these studies clearly underscore a critical need for agent that can effectively treat renal fibrosis, the final common pathophysiological event in the progression of CKD to ESRD.

Potential targets for combating inflammation and renal fibrosis in CKD are the nuclear farnesoid X receptor (FXR) and the soluble epoxide hydrolase (sEH) enzyme (13, 14). FXR expression is decreased in the human and rodent models of kidney disease and this decrease correlated with the level of inflammation and fibrosis in the kidney (13, 15). Activation of FXR demonstrated kidney protective actions in animal models by decreasing kidney inflammation, oxidative stress, and fibrosis (16, 17). FXR is expressed on immune cells and FXR activation has anti-inflammatory actions (17, 18). These findings indicated an important role for FXR in kidney disease. Similar to FXR activation, inhibition of sEH that metabolizes and inactivates kidney protective epoxyeicosatrienoic acids (EETs) can combat inflammation and kidney disease (14, 19, 20). Experimental studies demonstrated beneficial kidney actions of EETs and sEH genetic deletion or inhibition in animal models of renal fibrosis and CKD (19, 20). Interestingly, it has also been demonstrated that FXR activation can induce the expression of EET producing cytochrome P450 enzymes and several polyunsaturated fatty acids including arachidonic acid act as FXR ligands (21, 22). Intriguingly, our research group has developed a dual acting FXR agonist and sEH inhibitor, DM509 which could decrease renal inflammation and fibrosis that occurs in CKD (23, 24).

Previous studies have demonstrated in mice that DM509 protected against liver and kidney fibrosis (23, 25, 26). Our previous kidney study demonstrated that DM509 administered one day prior to unilateral ureteral obstruction (UUO) prevented development of renal fibrosis (26). Unfortunately, the clinical treatment of CKD occurs after significant renal inflammation and fibrosis are evident. Thus, in the present study DM509 was tested in a clinically relevant manner.

In the current study we investigated interventional treatment with DM509 to reduce renal inflammation and fibrosis to halt the progression of CKD. Our findings demonstrate the therapeutic potential for the dual acting agent DM509 to combat renal inflammation and fibrosis associated with CKD progression.

## Materials and methods

### Animal experiments

**Figure 1:**
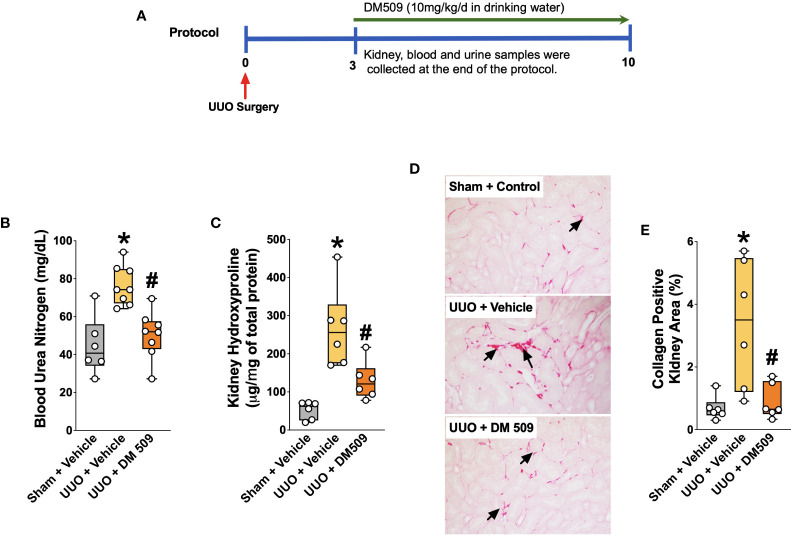
Experimental Protocol and DM509 Interventional Treatment on Renal Fibrosis in UUO Mice. Experimental protocol **(A)**; Blood urea nitrogen levels in experimental groups **(B)**; Kidney hydroxyproline levels in experimental groups **(C)**; Representative photomicrographs **(D)** depicting renal interstitial collagen (black arrows) and quantitative measurements of tubular interstitial collagen in experimental groups **(E)**. Data are reported as box and whisker plots with median and minimum to maximum, n=6. p<0.05, * UUO + Vehicle vs. Sham + Vehicle; # UUO + DM509 vs. UUO + Vehicle.

All animal experiments carried out in this study were approved and conducted according to guidelines of the Medical College of Wisconsin Institutional Animal Care and Use Committee guidelines. All mice were housed in the Biomedical Resource Center at the Medical College of Wisconsin with free access to water and food under a 12/12h light-dark cycle. Male C57Bl/6J mice (8-10 weeks) were purchased from Jackson Laboratories, Bar Harbor, ME. Mice were randomized in three groups (n=6 mice/group) and were subjected to Sham or UUO surgery to induce kidney disease. Prior to and during the surgical procedure, mice were administered 2.0% isoflurane to induce anesthesia. The UUO surgery was carried out by obstructing the left ureter proximal to the renal pelvis using a 6-0 silk tie (20, 26). Sham surgery was carried out in a set of mice using same procedure as the UUO mice except that the ureter was not ligated. In the present study we started DM509 interventional treatment 3 days after UUO as previous studies demonstrated robust renal inflammation and fibrosis on day 3 after UUO surgery (20). Mice were kept in separate cages to accurately measure fluid intake. Vehicle (hydroxypropyl methylcellulose (HPMC) and 0.01% Tween 80) and DM509 were administrated in drinking water and the correct daily dose was maintained by monitoring daily fluid intake. At the end of the 7-day treatment protocol, the mice were euthanized, and blood and kidney samples were obtained. The experimental protocol used in this study is shown in **Figure 1A**. Kidney samples for histological and immunohistological studies were fixed in 10% buffered formalin and stored at room temperature. Kidney tissue samples for biochemical and gene expression analysis were snap-frozen in liquid nitrogen and stored at -80°C until used.

### Biochemical analysis

Kidney hydroxyproline tissue levels were determined in tissue homogenates using a colorimetric assay kit (Catalog # MAK008, Sigma-Aldrich, USA). Protein content of the tissue homogenates was measured using Pierce™ BCA Protein Assay Kit (Catalog # 23225, Thermo Fisher Scientific, USA). Monocyte chemoattractant protein-1 (MCP-1) levels in kidney tissues were measured using ELISA (Catalog # BMS6005, Thermo Fisher Scientific, USA). Blood urea nitrogen (BUN) was measured using a colorimetric assay kit (Catalog # EIABUN, Thermo Fisher Scientific, USA).

### RNA isolation and real-time PCR analysis

Renal mRNA was isolated from kidney homogenates of each individual sample by RNeasy Mini Kit (QIAGEN, CA, USA) according to the manufacturer’s protocol. The purity and amount of mRNA in samples was quantified spectrophotometrically. For each sample, 1µg of total RNA was reverse transcribed to cDNA using iScript™ Select cDNA Synthesis Kit (Bio-Rad, Hercules, CA, USA). Real-Time (RT) PCR analysis was carried out using cDNAs to determine renal mRNA expression of the markers of fibrogenesis [fibronectin, α-smooth muscle actin (α-SMA), fibroblast specific protein-1 (FSP-1)], tubular injury [neutrophil gelatinase-associated lipocalin (NGAL), kidney injury molecule-1 (KIM-1), Claudin-1,-3 and -4], inflammation [tumor necrosis factor-α (TNF-α), interleukin-1β (IL-1β), and interleukin-6 (IL-6)], and vascular injury [intercellular adhesion molecule-1 (ICAM-1), vascular cell adhesion molecule-1 (VCAM-1), Claudin-5, and VE-Cadherin]. Gene expression was quantified by iScript One-Step RT-PCR Kit with SYBR green using the MyiQ^™^ Single Color RT-PCR Detection System (Bio-Rad Laboratories, Hercules, CA, USA). Dissociation curve analysis was done with iQ5 Optical System Software, Version 2.1 (Bio-Rad Laboratories, Hercules, CA, USA. During RT-PCR, denaturation was done at 95°C for 2 min followed by 40 cycles at 95°C for 10s and 30s at 60°C. Each sample was run in triplicate and fold change in gene expression compared to controls was calculated using the comparative threshold cycle (C_t_) method. The expression levels of the gene of interest were determined by normalizing C_t_ values to three housekeeping genes. All statistical analyses were carried out using 6 samples from each experimental group.

### Histopathology

Formalin-fixed kidney samples from each animal were paraffin embedded. Kidney samples were sectioned (5μm thickness), mounted on slides, and stained with Picrosirius Red (PSR) (Alfa Aesar, Tewksbury, MA). The stained slides were examined for interstitial fibrosis (PSR-collagen positive area) in the kidney at 200x magnification using NIS Elements AR version 3.0 imaging software (Nikon instruments Inc., Melville, NY, USA). Collagen-positive renal fibrotic area is presented as a percentage area-fraction relative to the total area. Histological analysis and the scoring of collagen-positive kidney area were performed by two observers in a blinded fashion.

### Immunohistopathological analysis of the kidney sections

Kidney histological slides prepared from the paraffin-embedded kidney tissue samples were deparaffinized and re-hydrated followed by overnight incubation with antibodies against α-SMA (1:100, Santa Cruz Biotechnology, USA), FSP-1 (1:50, Cell Signaling Technology, USA) and Epithelial-Cadherin (E-Cadherin, 1:100, Cell Signaling Technology, USA). Using similar experimental steps, kidney slides were incubated with antibodies against CD45 (1:100, Cell Signaling Technology) and CD31(1:50, Cell Signaling Technology). On the second day, slides were washed and incubated with biotinylated secondary antibody (1:200-1:300) for 45-60 minutes at room temperature. The presence of the target proteins in the kidney sections were determined from avidin-biotinylated HRP complex (Vectastain ABC Elite kit, Vector Laboratories, USA) followed by counterstaining with hematoxylin. Stained kidney slides were examined at 200x magnification with a light microscope and analyzed using Nikon NIS Elements Software (Nikon Instruments Inc., Melville, NY, USA). The renal area positive for a specific target protein was calculated by Nikon NIS Elements Software and expressed as the percentage area relative to total area examined. The analysis and scoring process was carried out in blinded fashion by two observers.

### Statistical analysis

GraphPad Prism^®^ Version 4.0 software was utilized to carry out one-way ANOVA followed by Tukey’s *post-hoc* test in order to establish statistical significance between groups (GraphPad Software Inc, La Jolla, CA, USA). All data are reported as box and whisker plots with median and minimum to maximum. A p value smaller or equal to 0.05 was considered significant.

## Results

### Interventional DM509 treatment attenuates renal fibrosis progression

**Figure 2:**
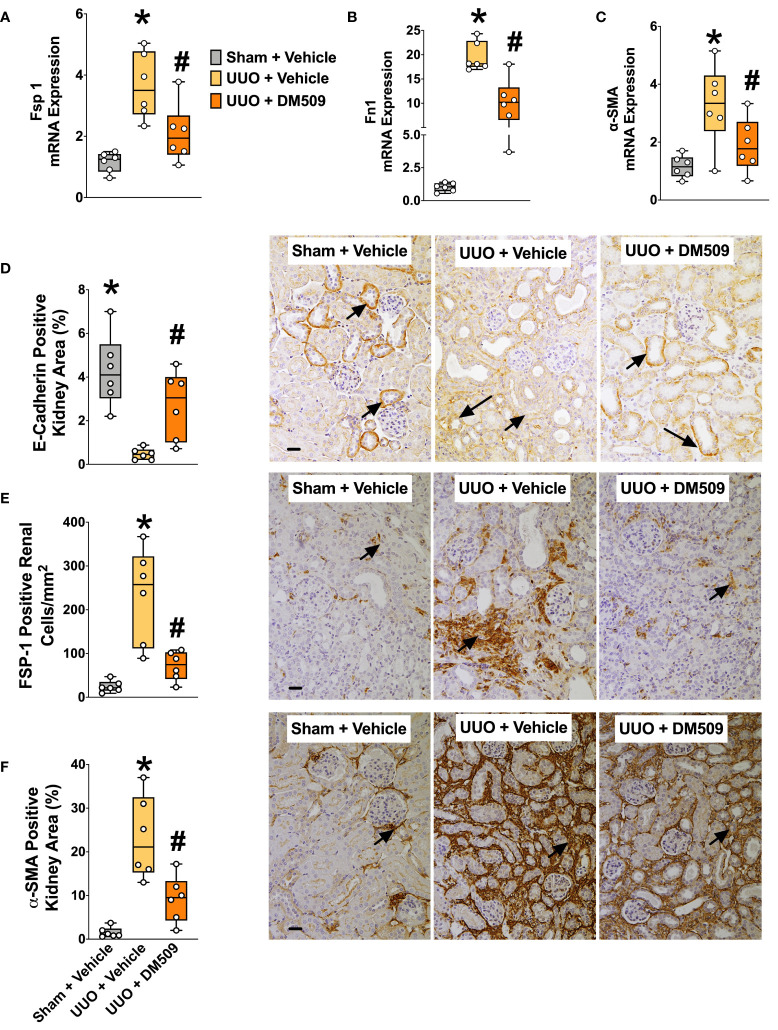
Attenuation of Renal Fibrosis Markers in UUO Mice by DM509 Interventional Treatment. Renal mRNA expression of fibrotic markers in experimental groups **(A–C)**; Quantitative measurement and representative photomicrographs for renal expression of FSP-1 **(E)** and α-SMA **(F)** in experimental groups; Quantitative measurement and representative photomicrographs showing renal expression of E-Cadherin **(D)** in experimental groups. Data are reported as box and whisker plots with median and minimum to maximum, n=6. p<0.05, * UUO + Vehicle vs. Sham + Vehicle; # UUO + DM509 vs. UUO + Vehicle.

Ten days following surgery, UUO mice exhibited renal injury with elevated BUN compared to the sham group. DM509 administered from day 3 to 10 reduced BUN by 40%. Mice with UUO developed marked renal fibrosis and demonstrated 5 times higher kidney level of hydroxyproline and 3 times higher collagen positive renal fibrotic area compared to sham mice. Dual FXR agonism-sEH inhibition with DM509 demonstrated marked anti-fibrotic effects and reduced kidney hydroxyproline content in UUO mice by 50% compared to UUO mice treated with vehicle. DM509 also reduced collagen positive fibrotic area in the kidney of UUO mice with levels like that in Sham mice (**Figures 1B–E**). Anti-fibrotic actions of DM509 were further assessed by evaluating renal mRNA protein expression of prominent fibrotic markers fibronectin, α-SMA and FSP-1 in UUO mice. We observed 3 to 19-fold higher mRNA expression of these fibrotic markers in the kidney of UUO compared to sham mice. In UUO mice, DM509 interventional treatment reduced renal mRNA expression of these markers by 40-50% compared to vehicle (**Figures 2A–C**). Renal expression of fibrotic markers was also determined at the protein level. UUO mice kidneys had nearly 90% higher expression of α-SMA and FSP-1 compared to sham mice. DM509 interventional treatment resulted in a robust 60-70% reduction of the renal expression of these markers in UUO mice compared to vehicle which further demonstrates a marked anti-fibrotic action (**Figures 2E, F**). An important mechanism of the fibrogenesis process is epithelial-to-mesenchymal transition (EMT). We observed higher renal expression of several mesenchymal markers including α-SMA and FSP-1 at both the mRNA and protein level in UUO mice. Renal expression of an important epithelial marker, epithelial-cadherin (E-Cadherin) demonstrated a robust 90% reduction in UUO compared to Sham mice. Interestingly, our findings demonstrate that DM509 interventional treatment reduced the loss of kidney E-cadherin levels in UUO mice kidney (**Figure 2D**). Overall, we demonstrate marked renal fibrosis and a fibrogenic process that involves EMT in UUO mice. We further demonstrated that interventional DM509 treatment has anti-fibrotic actions that are linked to a reduction in renal EMT in UUO mice.

### Renal inflammation is attenuated by DM509 interventional treatment in UUO mice

**Figure 3:**
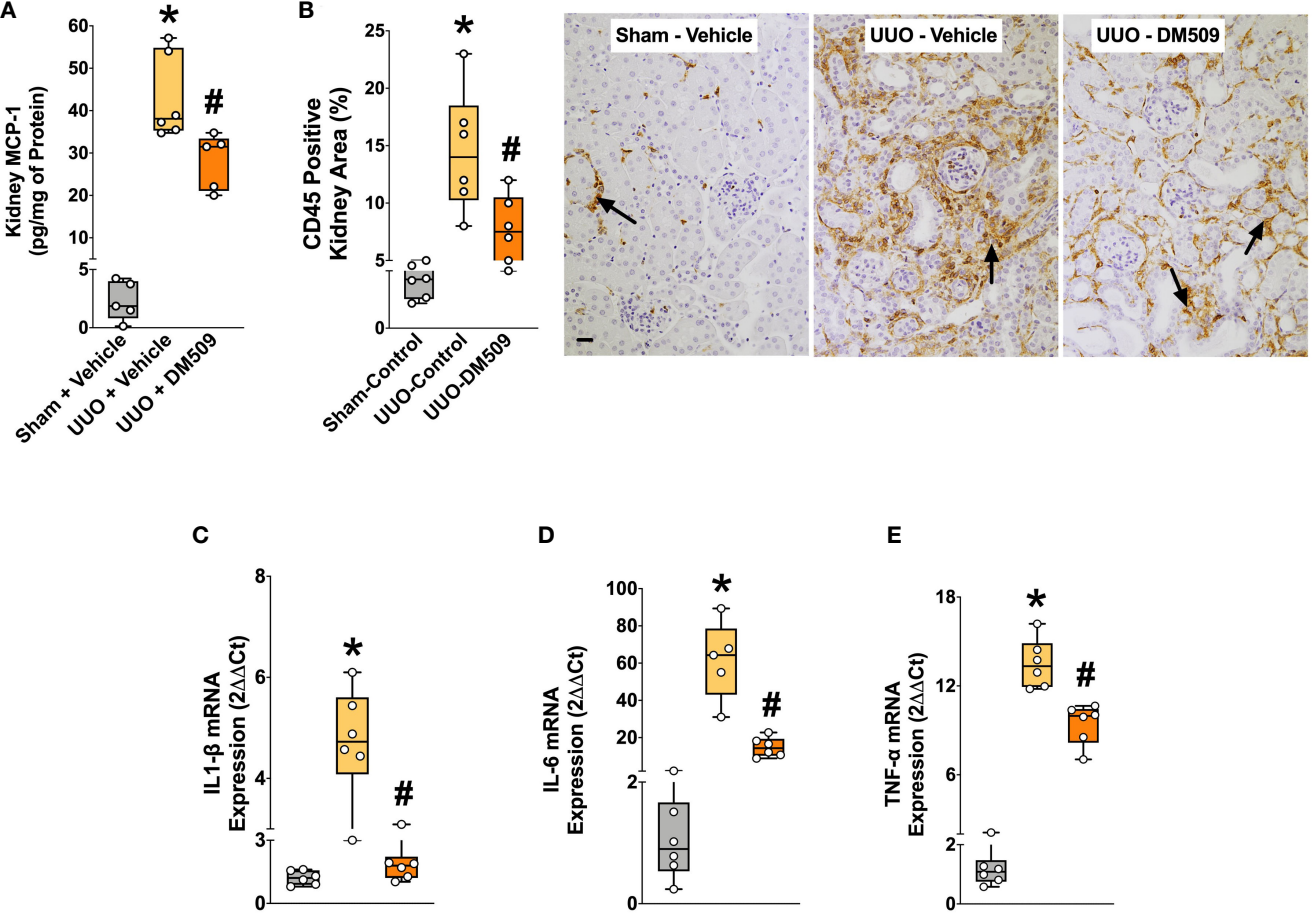
DM509 Interventional Treatment Reduced Renal Inflammation in UUO Mice. Kidney MCP-1 level in experimental groups **(A)**; Quantitative measurement of CD45 immunopositive kidney area **(B)** and representative photomicrographs showing CD45+ kidney area in experimental groups **(C)**; Renal mRNA expression of profibrotic cytokines in experimental groups **(D-E)**. Data are reported as box and whisker plots with median and minimum to maximum, n=6. p<0.05, * UUO + Vehicle vs. Sham + Vehicle; # UUO + DM509 vs. UUO + Vehicle.

In the present study, we demonstrate that UUO mice developed marked renal inflammation. Kidney content of an important chemokine, MCP-1 was 90% higher in the UUO mice compared to sham mice. DM509 interventional treatment reduced kidney MCP-1 levels by 35% in UUO mice (**Figure 3A**). UUO mice also demonstrated marked renal infiltration of CD45 positive immune cells as indicated by 80% higher kidney area that were positive for CD45 in UUO compared to sham mice. Interestingly, DM509 treatment markedly reduced renal infiltration of immune cells by 50% in UUO mice (**Figures 3B, C**). Considering a positive effect of DM509 on renal chemotaxis in UUO mice, we investigated renal mRNA expressions of several pro-fibrotic cytokines. We demonstrated a 5-60-fold higher renal expression of TNF-α, IL-6, and IL-1β mRNA in UUO compared sham mice. Interventional DM509 treatment reduced renal mRNA expression of these cytokines by 30-70% in UUO mice (**Figures 3D–E**). In summary, we demonstrated marked chemotaxis and higher levels of pro-fibrotic cytokines at mRNA level in the kidney of fibrotic UUO mice. Our findings also demonstrated that interventional DM509 treatment has anti-inflammatory actions on chemotaxis and profibrotic cytokine mRNA expression in the kidney of UUO mice.

### DM509 interventional treatment reduced renal tubular injury in UUO mice

**Figure 4:**
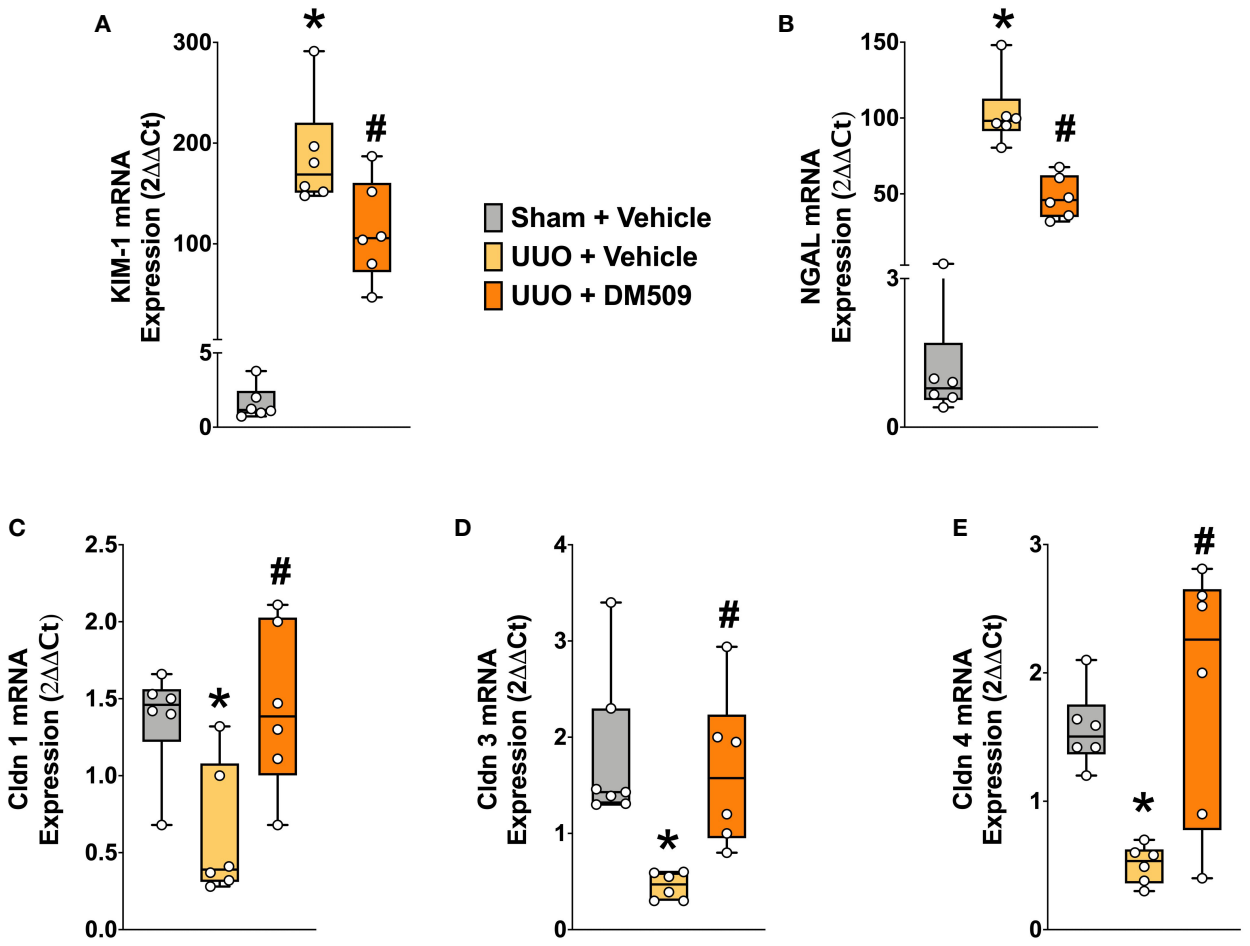
Renal Tubular Injury was Attenuated in UUO Mice by DM509 Interventional Treatment. Renal mRNA expression of tubular injury markers KIM-1 **(A)** and NGAL **(B)** in experimental groups; Renal mRNA expression of claudin 1,3 and 4 in experimental groups **(C-E)**. Data are reported as box and whisker plots with median and minimum to maximum, n=6. p<0.05, * UUO + Vehicle vs. Sham + Vehicle; # UUO + DM509 vs. UUO + Vehicle.

In renal fibrosis, tubular injury is a critical pathophysiological event and contributes to extracellular matrix formation. We demonstrated marked renal tubular injury in UUO mice with 100 to 150-fold higher mRNA expressions of KIM-1 and NGAL, two important tubular injury markers. Interventional DM509 treatment attenuated renal tubular injury and reduced mRNA expression of KIM-1 and NGAL by 40 to 50% in UUO mice (**Figures 4A, B**). Tubular injury was further assessed from the renal mRNA expressions of claudin-1, -3, and -4, tight junction proteins that are important for epithelial integrity. We demonstrated that renal mRNA expression of claudin-1, -3, and -4 was decreased by 50-75% in UUO mice compared to sham mice. Interestingly, Interventional DM509 treatment restored expression of claudins and brought renal mRNA expression levels in UUO mice to that in sham mice (**Figures 4C–E**). Overall, we demonstrated marked renal tubular injury in UUO mice which attenuated by interventional DM509 treatment.

### Renal vascular loss is attenuated by interventional DM509 treatment in UUO mice

**Figure 5:**
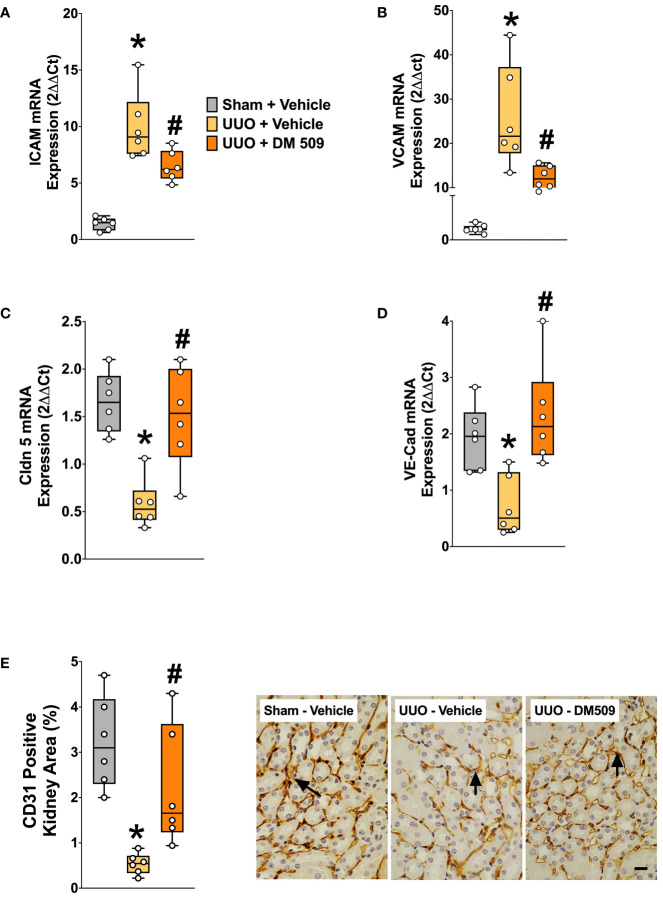
DM509 Interventional Treatment Reduced Renal Vascular Inflammation and Vascular Loss in UUO Mice. Renal mRNA expression of adhesion molecules ICAM **(A)** and VCAM **(B)** in experimental groups Renal mRNA expression of VE-Cadherin **(C)** and claudin 5 **(D)** in experimental groups; Quantitative measurement and representative photomicrographs showing renal expression for CD31+ kidney area in experimental groups **(E)**. Data are reported as box and whisker plots with median and minimum to maximum, n=6. p<0.05, * UUO + Vehicle vs. Sham + Vehicle; # UUO + DM509 vs. UUO + Vehicle.

In the present study, we demonstrated that UUO mice have marked 10-20-fold higher renal mRNA expression of vascular adhesion molecules ICAM and VCAM compared to sham mice. In UUO mice, interventional DM509 treatment reduced renal mRNA expression of these vascular adhesion molecules by 50-60% (**Figures 5A, B**). Renal mRNA expression of tight junction proteins VE-Cadherin and Claudin-5 that are important for endothelial integrity were significantly reduced by 60-70% in the kidney of UUO compared to Sham mice. Interestingly, DM509 treatment markedly attenuated the renal decrease of VE-Cadherin and Claudin-5 mRNA in UUO mice and the expression of these genes was restored to levels similar to sham mice (**Figures 5C, D**). Most importantly, we observed a marked 80% decrease in the renal vasculature as assessed by CD31 immunopositive area in the UUO mice kidneys. We further demonstrated that DM509 interventional treatment reduced renal vascular loss in UUO mice by restoring renal vasculature to a level similar to sham mice and CD31 positive kidney area of DM509 treated animals was 75% higher than vehicle treated UUO mice (**Figure 5E**). In summary, UUO mice exhibited marked renal vascular loss along with elevated expression of vascular adhesin molecules and reduced expression endothelial tight junction protein. Interventional DM509 treatment markedly reduced vascular loss in the kidney of UUO mice.

## Discussion

Fibrosis, a characteristic of all chronic kidney diseases (CKD), is recognized to be an independent predictor of disease progression (1, 3, 27). CKD and ESRD are major public health problems with increasing prevalence worldwide and in the US almost 15% (37 million) people are suffering from CKD (28, 29). Unfortunately, at present, there are no effective therapies to prevent or slow the progression of renal inflammation and fibrosis, and the treatment options for patients with ESRD are limited to dialysis and renal transplant (4–6). The lack of novel drugs targeting the pathological events of renal inflammation and fibrosis often give mixed results with unwanted side effects (5, 6). The shortcomings of these experimental therapies are likely due to the fact that the pathogenesis of fibrosis is complex. Renal fibrosis is linked to expansion of the interstitium with activated myofibroblasts, elevated inflammation, tubular atrophy, and microvascular injury (2, 30). In previous study our laboratory demonstrated that DM509 administered in a preventive manner reduced renal fibrosis in UUO mice (26). A major shortcoming of the previous study is that the clinical treatment CKD takes place after renal damage is evident. The current study administered DM509 to UUO mice at three days where renal inflammation and fibrosis is established (19, 20). DM509 is a dual acting FXR agonist and sEH inhibitor that possesses low nanomolar dual potency (31). Previous studies have demonstrated that following a single 10 mg/kg dose of DM509 to mice results in a robust increase in the EET/DHET (sEH substrate product) ratio and modulation of FXR regulated gene expression in the liver (31). In the present study we have demonstrate the unique ability of DM509 given in an interventional mode to reduce renal inflammation and slow progression of renal fibrosis in the mouse UUO model. Our findings demonstrate promising anti-inflammatory and anti-fibrotic effects of interventional DM509 treatment in regard to its ability to affect all renal fibrotic pathological events.

During renal fibrosis there is marked accumulation of myofibroblasts in the tubular interstitial space and these are the primary cells to synthesize and deposit pathological components of fibrillar matrix namely collagen and fibronectin (30, 32). Accumulation of fibrotic matrix progressively destroys the normal kidney tissue architecture by contraction and increased stiffness, resulting in disrupted blood flow supply and nephron function, as well as by increasing the space between vascular and tubular structures (30, 32). In the present study, we have used an established UUO mouse model that demonstrates marked renal fibrosis with accumulation of activated myofibroblast and collagen formation. These critical fibrotic events are evident from elevated renal collagen level and high expression of activated myofibroblast markers α-SMA and FSP-1. Interventional treatment with the dual FXR agonist and sEH inhibitor, DM509 attenuated collagen and myofibroblast formation and demonstrated a robust anti-fibrotic effect in the UUO kidney. The observed anti-fibrotic ability of DM509 can be attributed to the biological actions of both FXR agonist and sEH inhibitor pharmacophores.

Several earlier studies reported marked anti-fibrotic actions of sEHi in animal models of CKD and renal fibrosis, including the mouse UUO model (19, 20, 33). Indeed, genetic or pharmacological sEH inhibition markedly reduced renal fibrosis and extracellular matrix protein formation in UUO mice by acting on multiple fibrotic pathophysiological events (19, 20). The other pharmacophore of DM509, FXR is highly expressed within the kidney, and studies have demonstrated that FXR activation can mitigate renal injury (13, 16). FXR levels were inversely correlated with CKD progression in mice and the degree of interstitial fibrosis in humans (34). FXR activation using obeticholic acid also decreased kidney inflammation and fibrosis in an ischemic reperfusion model of renal injury (34). It has been demonstrated that the renal expression of FXR is decreased in UUO mice (35). In the same study, it was also demonstrated that a novel FXR agonist EDP-305 reduced renal fibrosis (35). Consistent with these findings, we suggest that the anti-fibrotic actions of interventional DM509 treatment is due to both FXR agonism and sEH inhibition.

Renal fibrosis is complex process consisting of several mechanisms that ultimately leads to myofibroblast formation and extracellular matrix synthesis (36–38). Initial cell injury in response to UUO results in cell stress and necrosis which leads to release of damage-associated molecular patterns (DAMPs) leading to inflammation (36, 37). Several mechanisms including cellular activation, cellular proliferation, immune cell infiltration, EMT, mesothelial-to-mesenchymal transition (MMT), and endothelial-to-mesenchymal transition (EndoMT) are responsible for increasing the net pool of myofibroblast (37, 38). The current study evaluated the ability for interventional DM509 treatment to reduce EMT, inflammation and EndoMT in UUO mice.

EMT is one of several contributors to renal fibrosis in UUO mice (39, 40). Consistent with these findings, in the present study the renal fibrosis in UUO kidney was accompanied by EMT with a markedly reduced expression of epithelial marker E-cadherin and higher expression of mesenchymal markers α-SMA and FSP-1. UUO mice also demonstrated marked renal fibrosis with collagen formation and myofibroblast formation. It has been reported that the anti-fibrotic effect of the FXR agonist, EDP-305 could be associated with its ability to reduce mesenchymal expression and restoration of epithelial expression within the renal tubules (35). The ability of DM509 to also act as an sEH inhibitor to maintain and elevate epoxyeicosatrienoic acids (EETs) levels could contribute to the reduction in EMT and anti-fibrotic actions. EETs have strong cardiovascular and renal actions and are kidney protective with potent anti-fibrotic actions (14, 33). Interestingly, FXR agonist have the ability to affect the production of EETs from arachidonic acid by inducing CYP P450 (CYP) epoxygenases (21, 22). Indeed, it has been reported that in a liver fibrosis model FXR activation reprogrammed arachidonic acid metabolism by inducing CYP epoxygenase expression and EET production (21). The current study demonstrated that the anti-fibrotic actions of DM509 in UUO model is associated with a reduction in EMT. The anti-fibrotic action of DM509 was associated with its ability to reduce renal tubular EMT. Our findings suggest that the reduction in EMT induced by DM509 is due to FXR agonism, sEH inhibition and the important interactive relationship of these two DM509 pharmacophore activities.

During renal fibrosis, inflammation plays a key role in the activation of fibroblast and extracellular matrix formation in the kidney (2, 30, 41). Upon activation by profibrotic cytokines, resident interstitial fibroblasts progressively gain the myofibroblast phenotype, which was consistently seen in various CKD models including the UUO model (39, 42). Indeed, it was found that the monocytes/macrophages are the most abundant immune cells in most CKD models, and the presence of macrophages in human CKD biopsies is associated with tubular interstitial fibrosis and poor renal survival (43). In the present study, we demonstrated marked renal inflammation with elevated kidney MCP-1 levels and renal infiltration of immune cells in the UUO kidney. Elevated kidney chemokine levels and presence of immune cells in the UUO kidney were accompanied by elevated renal expression of key profibrotic cytokines. These findings are consistent with our earlier findings in the UUO renal fibrosis model (19, 20, 39). The dual acting molecule DM509 administered as an interventional treatment markedly reduced renal inflammation by reducing MCP-1 levels, infiltration of immune cells, and cytokine expression in UUO mice. The anti-inflammatory action of DM509 can be attributed to the biological actions of both FXR agonism and sEH inhibition activities. Anti-inflammatory actions of sEH inhibition in reducing kidney injury are well established in CKD animal models (19, 20, 44). Inhibition of sEH is known to reduce chemotaxis by reducing chemokines, renal infiltration of immune cells, and cytokine production in CKD (14, 20, 33). The sEH inhibitory anti-inflammatory actions in reducing renal fibrosis are also well established (14, 20). It has been reported that sEH inhibition either by genetic manipulation or by a pharmacological inhibition provides robust anti-inflammatory actions and attenuates renal fibrosis progression in UUO mice (19, 20). In UUO mice, sEH inhibition resulted in a marked decrease in renal infiltration of different types of immune cells and reduced profibrotic cytokine production which are linked to renal fibrotic pathophysiology (19, 20). The increase in EET levels in response to sEH inhibition could have direct actions on immune cells. A recent study provided evidence that renal EETs may act locally to regulate the ENaC activity in circulating monocytes entering the kidney to reduce inflammation in salt-sensitive hypertension (45). FXR activation has also been shown to suppress the inflammatory response by affecting several critical aspects of inflammatory processes (13, 17). FXR activation inhibited inflammation in various organ systems including liver, lung, and kidney (17). In several studies using rodent models of type I diabetes, high-fat diet-induced cardiometabolic syndrome, and type 2 diabetes mellitus, FXR expression and its target genes were down regulated in the kidney (46–48). Interestingly, such changes in the renal expression of FXR and its target genes in these models with renal pathology correlated with elevated inflammation and fibrosis in the kidney (46–48). A role of FXR was also reported in the UUO renal fibrosis model where it was demonstrated that FXR activation reduced renal inflammation and fibrosis by decreasing renal immune cell infiltration and expression of profibrotic cytokines (35). Like sEH inhibition and increased EET levels, FXR could act directly on immune cells. Indeed, previous studies have demonstrated that FXR agonists inhibit gastrointestinal inflammation through direct actions on immune cells to preserve the intestinal barrier during inflammatory bowel disease (49). A link to sEH inhibition could also exists as the FXR mediated anti-inflammatory actions require active CYP epoxygenases (21). Overall, the well-known anti-inflammatory actions of sEH inhibition and FXR agonism indicate that the robust anti-inflammatory actions of the novel dual acting DM509 are associated with the biological consequences of both molecular activities.

Renal inflammation caused by elevated chemokines, infiltrating immune cells and cytokine productions cause tubular injury and this tubular injury is considered an important event in renal fibrosis and CKD progression (3, 30, 42). Indeed, tubular degeneration evokes a vivid peritubular environment, and the injured tubular cells initiate a local inflammation, that finally leads to the removal of all tubular remnants and the formation of fibrosis (30, 42). An interesting finding from a recent reversal UUO study found an important contribution for F4/80 positive macrophages but not CD3+ T-cells in salt-sensitive hypertension (44). Our research group has previously demonstrated that DM509 treatment to non-alcoholic steatohepatitis mice decreased liver CXCR3, CXCL9, CXCL10, TNFα, IL-1β, and TGF-β expression (23). In the current study we demonstrate a similar DM509 anti-inflammatory action in UUO. Kidney MCP- levels, CD45 positive immune cells, and TNFα, IL-1β, and IL-6 IL gene expression were decreased by DM509 treatment. Previous studies with sEH inhibitors or genetic deletion have demonstrated decreased transforming growth factor beta (TGF-β) in UUO mice (19, 20, 50). Likewise, FXR has been demonstrated to have anti-fibrotic actions in UUO mice via regulating TGFβ-Smad3 pathway (16, 34). FXR expression can be found on macrophages, tissue resident macrophages, and dendritic cells (18, 51). Future studies are necessary to evaluate the actions of interventional DM509 treatment on TGF-β and specific immune cells. Nevertheless, TGF-β is a critical regulator that is likely inhibited by DM509 to decrease myofibroblast activation, renal fibrosis, and tubular injury.

In the present study, we demonstrated tubular injury with elevated renal KIM-1 and NGAL expression. These findings are consistent with findings of a recent study with the UUO renal fibrosis model (26). The current study also demonstrated reduced expression of claudins in the UUO mice. Claudins exhibit a specific expression pattern in the kidney and are considered to be essential in tight junction formation to establish close connections between epithelial cells, thereby maintaining cell polarity and tubular integrity (52, 53). Loss of tubular integrity and polarity of the tubular epithelial cells are associated with tubular epithelial injury and renal fibrosis (53, 54). Interventional treatment with dual acting FXR agonist and sEH inhibitor, DM509 reduced renal tubular injury and restored tubular epithelial integrity in UUO mice. We suggest that this DM509 action is a consequence of both FXR agonism as well as sEH inhibition. Several studies have demonstrated that sEH inhibition can reduce renal tubular injury (14, 19, 20). A similar renal tubular protective effect of sEH inhibition has also been reported in ob/ob mice with diabetic nephropathy and renal fibrosis (55). These findings indicate that sEH inhibitory activity byDM509 protects renal tubules. FXR agonism has also demonstrated to be renal tubule protective in several studies (34, 56). Obeticholic acid, a FXR agonist provided renal protection with reduced renal inflammation, tubular injury, and fibrosis in a lipopolysaccharide-induced acute kidney injury model (56). The FXR agonist obeticholic acid demonstrated a similar renal tubular protective and anti-fibrotic effect in a rodent model of renal ischemia injury (34). The findings of the current study demonstrate that interventional treatment with the dual FXR agonists and sEH inhibitor, DM509 acts through combined reduction in inflammation, attenuation of EMT, and decreased tubulointerstitial fibrosis significantly decreased renal injury in UUO mice.

Similar to renal tubular injury, another important pathophysiological event in CKD progression is vascular injury followed by vascular loss (56, 57). Intact microvasculature is a prerequisite for normal tubular structure and functions and peritubular vascular injury is associated with CKD progression (56–58). Indeed, a decrease in CD34+ tubulointerstitial capillaries has been observed together with tubular interstitial fibrosis in the UUO model (59). In a recent study, we demonstrated marked renal vascular loss in the UUO kidney with a decrease in CD31+ renal vasculature (50). Consistent with these previous findings, the current study demonstrated marked renal vascular loss in UUO mice with reduced renal CD31+ vascular levels. UUO mice also demonstrated kidney vascular inflammation with elevated expression of adhesion molecules ICAM and VCAM and a leaky endothelial barrier with elevation in claudin-5 and VE-cadherin. Claudin-5 and VE-Cadherin are specifically expressed in endothelial cells to maintain vascular barrier functions and regulate endothelial permeability (60, 61). In the UUO model reduction of renal Claudin-5 and VE-Cadherin was demonstrated to be associated with renal fibrosis progression (62). Interestingly, in the present study the dual acting molecule DM509 reduced CD31+ renal vascular loss in UUO mice. DM509 interventional treatment also reduced renal vascular inflammation and endothelial permeability in the UUO kidney by reducing ICAM and VCAM expression and also by attenuating the loss of claudin-5 and VE-Cadherin.

It is important to note that in the UUO kidney, we demonstrated markedly higher expressions of mesenchymal markers α-SMA and FSP-1 and reduced expression of endothelial markers VE-cadherin and CD31. This difference in the expressions of endothelial and mesenchymal markers indicate a possible endothelial-to-mesenchymal transition (EndoMT) in the UUO kidney. The EndoMT is a complex process by which certain endothelial cell subsets lose endothelial characteristics and transform into mesenchymal or smooth muscle cells (63, 64). EndoMT is often linked to the development of fibrosis in animal models, including the UUO model (65). We suggest that the effects of DM509 on the renal vasculature in UUO mice is caused by both its FXR agonistic property and ability to inhibit sEH. FXR activation by obeticholic acid, a synthetic FXR agonist, attenuated vascular remodeling of pulmonary vasculature in a rat model of pulmonary hypertension (66). This study suggested that FXR activation mediated anti-inflammatory actions to reduce EndoMT (66). Likewise, sEH inhibition has been demonstrated to stabilize EET levels, inhibit vascular inflammation, and promote vascular repair through neovascularization (14, 67, 68). An increase in EET levels induced by treatment with the sEH inhibitor 12-(3-adamantan-1-yl-ureido)-dodecanoic acid (AUDA) promoted vascular repair in human coronary arterial endothelial cells (68). Pharmacological sEH inhibition also reduced coronary artery inflammation in a mouse model of heart disease associated with coronary artery aneurysms and myocardial infarction (68). Similar to AUDA, another sEH inhibitor t-AUCB dose dependently increased the expression of the angiogenic factor vascular endothelial growth factor (VEGF) (67, 69). Overall, we demonstrated that the novel dual acting molecule DM509 given as an interventional treatment reduced vascular loss during renal fibrosis by reducing vascular inflammation, restoring renal vasculature, and restoring vascular endothelial integrity. Our data also indicated a possible role of DM509 on EndoMT to reduce renal vascular injury in UUO mice.

## Conclusion

We demonstrated renal anti-fibrotic actions of a novel first-in-class dual acting molecule DM509 that simultaneously act as a FXR agonist and sEH inhibitor. Interventional treatment with DM509 reduced renal fibrosis in UUO mice by acting on several critical inflammatory mediated pathophysiological events. Our data demonstrated that DM509 uniquely reduced extracellular matrix formation and renal tubular and vascular injury in UUO mice. Our data also suggest that DM509 anti-inflammatory and anti-fibrotic actions are associated with its ability to reduce renal EMT and EndoMT. Overall, these data reveal that DM509 given in a clinically relevant manner is a promising dual acting anti-fibrotic and anti-inflammatory therapeutic that attenuates renal epithelial and vascular pathological events associated with progression of CKD to ESRD.

## Data availability statement

The raw data supporting the conclusions of this article will be made available by the authors, without undue reservation.

## Ethics statement

The animal study was approved by Medical College of Wisconsin Institutional Animal Care and Use Committee. The study was conducted in accordance with the local legislation and institutional requirements.

## Author contributions

MK: Conceptualization, Data curation, Formal analysis, Investigation, Methodology, Validation, Writing – original draft, Writing – review & editing. BN: Data curation, Formal analysis, Investigation, Methodology, Writing – review & editing. AS: Data curation, Formal analysis, Investigation, Methodology, Writing – review & editing. DM: Conceptualization, Data curation, Formal analysis, Funding acquisition, Investigation, Resources, Writing – review & editing. JI: Conceptualization, Funding acquisition, Resources, Supervision, Writing – original draft, Writing – review & editing.
